# Upper-Extremity Deep Venous Thrombosis following a Fracture of the Proximal Humerus: An Orthopaedic Case Report

**DOI:** 10.1155/2019/6863978

**Published:** 2019-11-04

**Authors:** John Strony, Gerard Chang, James C. Krieg

**Affiliations:** ^1^The Rothman Orthopaedic Institute Department of Research, Sheridan Building 10th Floor, 125 S 9th Street, Philadelphia, PA 19107, USA; ^2^Department of Orthopaedic Surgery, Thomas Jefferson University Hospital, 132 S 10th Street, Philadelphia, PA 19107, USA; ^3^The Rothman Orthopaedic Institute, 925 Chestnut Street, 5th floor, Philadelphia, PA 19107, USA

## Abstract

Deep venous thrombosis of the lower extremities following orthopaedic surgery is well-documented. Though less common than its lower extremity counterpart, upper extremity deep venous thrombosis (UEDVT) has been documented in the literature as well, largely in the context of arthroscopic shoulder surgery. However, there is a paucity of literature documenting UEDVT following surgical fixation of upper extremity fractures, specifically fractures involving the proximal humerus. We present a case of UEDVT following a fracture to the proximal humerus and subsequent surgery. Though UEDVT is considered a rare complication following this type of surgery based on a lack of documentation within the literature, we believe a high-index of suspicion is required to prevent potentially life-threatening sequelae, such as pulmonary embolism (PE) and post-thrombotic syndrome.

## 1. Introduction

Deep venous thrombosis (DVT) is the formation of a thrombus within the deep veins of the upper or lower extremity. Though more common within the lower extremities, DVT can occur in any of the veins of the upper extremity or thoracic inlet, including the jugular, brachiocephalic, subclavian, axillary, and brachial veins [1]. The occurrence of symptomatic upper extremity DVT (UEDVT) subsequent to arthroscopic shoulder surgery has been well-documented [2, 3]. However, with the exception of a few case reports [4], there is scarce literature regarding the development of UEDVT following the fracture of the upper extremity. Therefore, we report a case of UEDVT following surgical management of a proximal humerus fracture and repair of rotator cuff.

## 2. Case Presentation

**Figure 1:**
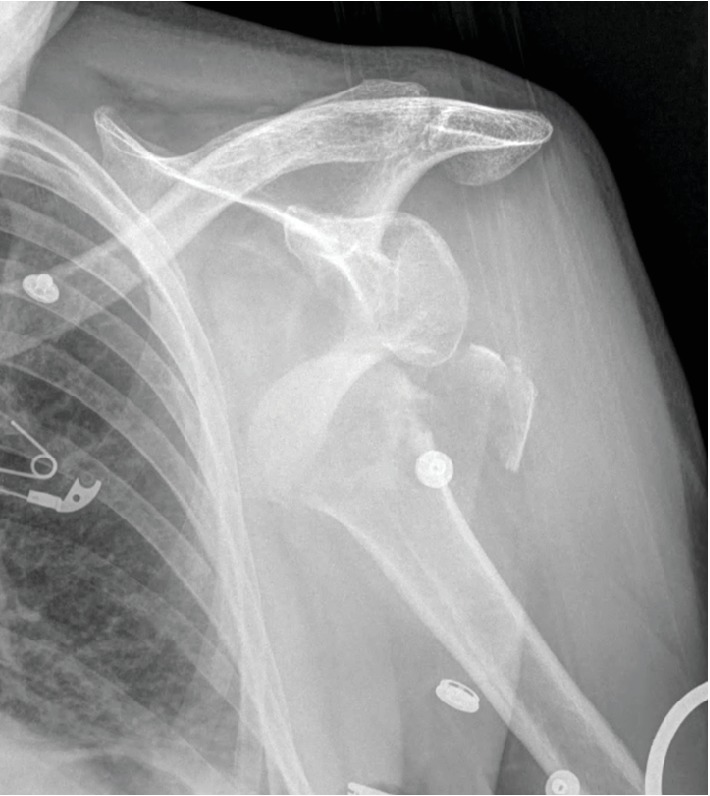
Four-part fracture of the left proximal humerus. A plain radiograph of the left shoulder taken in the anteroposterior (AP) direction at presentation showing a displaced, four-part fracture of the proximal humerus with involvement of the surgical neck and greater and lesser tuberosities.

**Figure 2:**
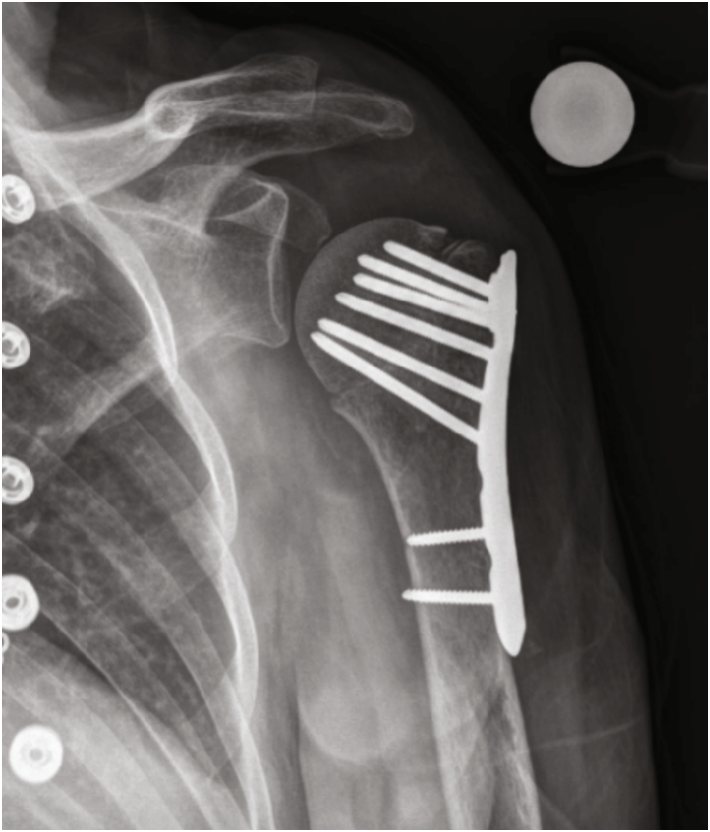
Left proximal humerus fracture following open reduction and internal fixation. A plain radiograph of the left shoulder taken in the anteroposterior (AP) direction six weeks after surgery. There is no displacement of the fracture fragments and reduction is satisfactory.

A 66-year old right hand dominate female, with no significant past medical history, presented to the emergency department at our institution with left shoulder pain after an accidental trip and fall onto an outstretched hand that occurred the same day. She had noticeable deformity of the upper arm and was neurovascularly intact distally. Radiographs were obtained which demonstrated a four-part proximal humerus fracture and dislocation of the left glenohumeral joint (Figure 1). The patient was admitted, and on hospital day 3, she underwent open reduction internal fixation of her proximal humerus fracture dislocation (Figure 2). A deltopectoral approach was utilized for this procedure. Venous bleeding was controlled with electrocautery. The cephalic vein was properly identified during the dissection and was not injured. There were no other intraoperative complications. Postoperatively, the patient had an uneventful hospital course and was discharged on postoperative day two on 325 mg acetylsalicylic acid once daily for DVT prophylaxis. On postoperative day five the patient returned to our outpatient clinic complaining of increased swelling and discomfort of the left upper extremity. Physical exam showed increased swelling with pitting edema and an intact neurovascular exam. An upper extremity duplex ultrasound was obtained which demonstrated occlusive thrombosis in the cephalic and deep brachial veins of the left forearm. The patient was referred to our Vascular Medicine service and anticoagulation therapy was initiated with enoxaparin 40 mg subcutaneously once daily for two days followed by rivaroxaban 15 mg twice daily for 21 days. Follow-up examination demonstrated improvement in symptoms and reduction in swelling. The patient was then transitioned to long-term prophylaxis using rivaroxaban 20 mg daily for eight weeks. A repeat upper extremity duplex ultrasound was obtained showing resolution of the DVT. She completed the remainder of her therapy with aspirin 81 mg daily for 16 weeks at which time her symptoms had completely resolved. The patient did not experience any recurrent thromboembolic events or any complications from her anticoagulation therapy. Of note, the patient continued her routine postoperative rehabilitation program with early passive range of motion followed by gradual transition to active range of motion and strength training with physical therapy. In addition, postoperative radiographs were obtained throughout her follow up which showed continual consolidation of the fracture site with complete bony union at seven months.

## 3. Discussion

DVT following orthopaedic surgery is well documented within the literature. In particular, much is known about DVT after reconstructive hip, knee, and shoulder arthroplasty [3, 5] as well as after surgery for long bone metastasis [6] and acute fracture repair of the lower extremities [7]. However, there is a paucity of literature addressing the incidence, characterization, and long-term outcome of UEDVT following surgical fixation of upper extremity fractures. Overall, UEDVT has been rarely reported with an incidence of 0.4-1 case per 100,000 patients [8].

The pathogenesis of UEDVT can be divided into either primary or secondary causes. Primary causes are less common and include venous thoracic outlet syndrome, Paget-Schroetter syndrome, and idiopathic causes. Paget-Schroetter related thrombosis occurs in individuals who develop muscle hypertrophy and vessel wall damage following strenuous exercise in the context of narrowed thoracic inlet [9]. Secondary causes include the presence of an indwelling device or underlying pathology that predisposes the patient to vascular thrombosis. The most frequent etiology of UEDVT of a secondary nature is indwelling central venous catheter placement. Recently, the increased use of peripherally placed central venous catheters and associated patient co-morbidity has witnessed a rise in UEDVT and can represent up to 10% of all DVT cases. It is estimated that the placement of central venous catheters accounts for up to 80% of UEDVT. Other secondary causes include previously placed pacemaker leads, cancer-associated thrombosis, and surgery involving the upper extremity [8].

We believe that the formation of an UEDVT in this patient was directly related to her proximal humerus fracture and subsequent surgery with immobilization. Blunt trauma increases the circulation of proinflammatory cytokines and procoagulant microparticles, creating a hypercoagulable state [10]. In addition, localized swelling occurs after surgery, leading to venous stasis. This patient did not have a personal history of malignancy or previous cardiac pacemaker nor were any central lines placed during her brief hospitalization. She did not demonstrate any anatomic abnormalities at the costoclavicular junction via various imaging modalities nor did she recall any activity that resulted in repetitive external rotation or abduction at the shoulder joint, lowering the suspicion of venous thoracic outlet syndrome or Paget-Schroetter syndrome [9].

The signs and symptoms of UEDVT are relatively nonspecific such as swelling, pain, edema, and erythema, regardless of the structure involved [11]. As symptoms are frequently limited to pain and swelling, the diagnosis of DVT can be easily overlooked and can be attributed to the natural course of the muscle injury and surgical repair that occurred. The differential diagnosis should consider cellulitis and lymphedema. Evaluation should initially include doppler ultrasound because of a reported sensitivity and specificity of 84% and 93%, respectively [12]. Limitations of doppler ultrasound include anatomical shadowing and obliteration by implanted devices. MRI and CT angiography may also be considered. Both modalities require contrast administration and its associated risks and carry similar implant-related imaging limitations. Contrast venography is rarely performed due to its invasive nature, its discomfort to the patient, and its use of ionizing radiation [12].

Anticoagulation is the mainstay of a multimodal approach to treatment for DVT. Goals of treatment include reducing further clot propagation, preventing embolization and promoting resolution of the existing thrombus. As such, patients should be started on a parenteral anticoagulant upon diagnosis. Such parenteral anticoagulants include unfractionated heparin or LMWH [13]. Following initial treatment with a parenteral anticoagulant, the patient should be transitioned to long-term oral anticoagulation. Current American College of Chest Physicians ACCP Guidelines recommend therapy for three months with oral anticoagulation by using either a direct oral anticoagulant or a vitamin K antagonist [13]. If warfarin is used the goal is to maintain an INR level of 2-3. Because of the proximity of surgery to the onset of DVT, thrombolysis should be reserved for individuals with greatest risk of complications; in all cases a standard course of anticoagulation should be continued following successful thrombolysis [13]. Thrombolysis should not be used in individuals with active bleeding, history of stroke or neurosurgery within previous two months, or surgery within the preceding ten days. Thrombolysis may be delayed but is most effective when instituted within several weeks of symptom onset. Surgical correction is reserved for those individuals with an external source of venous compression, typically primary UEDVT. In cases of recurrent UEDVT or pulmonary essmbolism (PE) resulting from UEDVT, the placement of a superior vena cava filter has been reported. However placement of such a filter is not widely recommended due to the lack of evidence demonstrating efficacy [13].

Adequate treatment of UEDVT is absolutely critical to prevent possible life-threatening sequelae, including PE. Symptomatic pulmonary emboli occur in approximately 12% of all UEDVT cases while asymptomatic pulmonary emboli occur in an additional 36% of such cases [14]. Additional long-term complications include post-thrombotic syndrome, the most common chronic complication following DVT. It is the result of outflow obstruction secondary to thrombus formation. It typically presents with chronic limb pain, swelling, and possible ulcer formation [15].

## 4. Conclusion

Although uncommon, UEDVT can occur following surgical fixation of an upper extremity fracture. Sequelae, such as PE and post-thrombotic syndrome, necessitate the need for a high index of suspicion. Persistent or worsening symptoms of swelling and pain should trigger a diagnostic workup consisting of doppler ultrasound imaging. Because pain and swelling are almost universal, to some degree, after all cases of surgical treatment of a proximal humerus fracture, we raise this issue so that clinicians continue to keep UEDVT in the differential diagnosis when swelling seems more excessive than normal, or when the patient presents with significant concern.

If a diagnosis is confirmed then prompt treatment with systemic anticoagulation should be initiated and continued for three months. The risk of recurrence and possible life-threatening sequelae warrant consideration for a multimodal approach to treatment if symptoms do not promptly resolve.

This case report highlights the risk of UEDVT as a complication following surgical fixation of a proximal humerus fracture. Knowledge of this complication along with close vigilance of post-operative symptoms and clinical course should trigger a diagnostic work up of UEDVT followed by appropriate therapy.
